# HSV-2 gC2 mRNA immunization in mice protects by producing antibodies that bind immune evasion epitopes

**DOI:** 10.1371/journal.ppat.1014337

**Published:** 2026-06-25

**Authors:** Lauren M. Hook, Tina M. Cairns, Sita Awasthi, Kevin P. Egan, Manaswini Gopalakrishnan, Zauraiz Syeda, Norbert Pardi, Tomas Bergstrom, Gary H. Cohen, Harvey M. Friedman

**Affiliations:** 1 Infectious Disease Division, Department of Medicine, Perelman School of Medicine, University of Pennsylvania, Philadelphia, Pennsylvania, United States of America; 2 Department of Basic and Translational Sciences, School of Dental Medicine, University of Pennsylvania, Philadelphia, Pennsylvania, United States of America; 3 Department of Microbiology, Perelman School of Medicine, University of Pennsylvania, Philadelphia, Pennsylvania, United States of America; 4 Department of Infectious Diseases, University of Gothenburg, Gothenburg, Sweden; University of Zurich, SWITZERLAND

## Abstract

Our vaccine candidate for genital herpes includes three immunogens involved in virus entry and immune evasion. HSV-2 glycoprotein C (gC2) is one of the immune evasion molecules that inhibits complement activation by binding C3b, and is the focus of this manuscript. Mice were immunized with 0.25, 0.5, 1, 10, or 30 µg of gC2 lipid nano particle (LNP)-encapsulated nucleoside-modified mRNA and challenged intravaginally with HSV-2. The gC2 mRNA-LNP, even at the lowest dose, was highly protective as a single immunogen. We measured antibody responses to six gC2 epitopes. Both neutralizing and C3b binding epitopes on gC2 were targeted. We passively immunized mice with monoclonal antibodies (mAbs) to gC2 and determined that the mAbs that enhance complement activation by blocking C3b binding were protective, while the mAb that neutralizes the virus, but does not block C3b binding, failed to protect. These results highlight the importance of a vaccine immunogen that induces antibodies that block the ability of gC to inhibit complement activation.

## Author summary

Our goal is to develop a vaccine to prevent genital herpes. Our strategy is to include important viral proteins in the vaccine that enable entry, but also viral proteins that dampen host immune responses. Often, entry proteins like glycoproteins B and D of HSV-2 (gB2, gD2) are selected for vaccine formulations because antibodies that bind to important entry epitopes can block the virus from infecting cells. Proteins that aid viruses in dampening host immune responses (immune evasion) have largely been overlooked in vaccine development, despite their contribution to virulence. We selected gC2 as one of our vaccine immunogens because it dampens host immune responses by binding complement component C3b. We first mapped C3b binding and neutralizing epitopes on gC2 and showed that mice that had been immunized with gC2 mRNA-LNP produced high titers of antibodies to these epitopes and were protected after an intravaginal HSV-2 challenge. We then passively immunized mice with mAbs to gC2 and determined that mAbs that recognize C3b binding epitopes were protective, while a mAb that neutralizes virus, failed to protect. These results highlight the importance of a vaccine strategy aimed at blocking immune evasion of HSV-2.

## Introduction

Herpes simplex virus type 2 (HSV-2) genital infection causes painful recurrences, predisposes individuals to HIV infection, and poses a risk of transmission to intimate partners and maternal transmission to newborns [1–4]. A vaccine to prevent HSV-2 genital infection is greatly needed, yet to date, none has been FDA-approved. The largest vaccine trials to prevent genital herpes in humans have involved subunit antigen vaccines aimed at blocking virus entry via antibodies produced to HSV-2 glycoprotein D (gD2) or gD2 and glycoprotein B (gB2) [5–7]. These vaccine efforts failed to achieve their primary endpoints of preventing genital herpes lesions or preventing both genital lesions and evidence of subclinical (latent) infection.

Antibodies are key correlates of protection for nearly all successful prophylactic vaccines and will likely be key to the success of a prophylactic genital herpes vaccine [8,9]. Antibodies to HSV acquired through infection protect neonates from acquiring HSV during delivery via placental transfer and reduce the severity of infection in individuals with prior oral HSV-1 infection who later acquire genital HSV-2 infection [10–12]. Antibodies produced following vaccination in the Herpevac Trial for Women correlated with protection against vaginal HSV-1, and HSV-specific IgG protects in preclinical animal models of genital infection [13,14]. To be effective, however, antibodies must be sufficiently potent and durable, and they must target crucial functional epitopes.

Vaccine efforts to prevent genital herpes have been hampered by low neutralizing antibody titers or low antibody-dependent cellular cytotoxicity titers and poor durability [5–7,15]. One possible explanation is that HSV-2 evades antibody-mediated immunity. Antibodies that are raised to HSV-2 entry molecules gB2 or gD2 through vaccination are rendered less effective by two immune evasion molecules encoded by HSV-2. HSV-2 glycoprotein C (gC2) binds complement component C3b, which inhibits complement activation and blocks complement-dependent neutralization of the virus [16,17]. HSV-2 glycoprotein E (gE2), as a heterodimer with HSV-2 glycoprotein I (gI2), binds the Fc domain of an IgG molecule to block effector functions mediated by the IgG Fc domain, including complement activation [18,19]. Another possible explanation is that crucial functional epitopes are often hidden from host immunity by carbohydrate shields or by neighboring proteins and require optimal vaccine formulations to render them immunogenic [20,21].

To address these challenges, we designed a vaccine candidate aimed at blocking viral entry by targeting gD2, as in previous vaccine trials, and blocking immune evasion of HSV-2 by targeting gC2 and gE2. Like gD2, both gC2 and gE2 are expressed on the virion envelope and on the surface of infected cells, where they are potential targets of antibodies produced through vaccination. Our vaccine candidate, a trivalent lipid nanoparticle (LNP)-encapsulated nucleoside-modified mRNA vaccine consisting of gC2, gD2, and gE2, elicited high antibody titers to all three vaccine antigens and was highly protective and durable in preclinical mouse and guinea pig models [9,22]. Antibodies that correlate strongly with protection were produced to crucial gD2 epitopes [9,23]. Importantly, the trivalent mRNA vaccine also produced high titers of antibodies to gC2 and gE2 that blocked them from interacting with C3b and the Fc domain of IgG, respectively, suggesting that the mRNA vaccine is likely targeting key functional epitopes on these glycoproteins as well [9]. A nucleoside-modified mRNA-LNP vaccine candidate that contains these three immunogens is currently in phase 1 human trials (BNT163, ClinicalTrials.gov ID NCT05432583).

Determining correlates of protection in preclinical studies for the individual immunogens in the trivalent mRNA vaccine will be an important aid in predicting vaccine success in humans. Correlates of protection studies performed in a guinea pig study demonstrated that the number of epitopes recognized by gD2-vaccinated guinea pigs positively correlated with protection against genital lesions. The importance of each crucial epitope was confirmed by passive transfer of individual mAbs that improved survival and reduced genital disease in mice after HSV-2 genital challenge [24]*.* The epitopes of gD2 function complementarily by interacting with nectin-1, herpes virus entry molecule (HVEM), or both viral entry receptors, and sequentially trigger the fusion machinery, providing a mechanistic explanation for the observed protection [25].

The aim of our study was to identify and characterize antibody correlates of protection to the crucial functional epitopes of gC2. We assembled a panel of mouse mAbs specific to gC2 and arranged them into antigenic communities using high-throughput biosensor technology. We then assigned biological function to each community, determining which targeted neutralizing epitopes, C3b-binding epitopes, or neither. Female BALB/c mice that had been immunized with increasing doses of a nucleoside-modified mRNA vaccine encoding the ectodomain of gC2, packaged within LNPs, were then evaluated for antibody responses, including those to important functional epitopes on gC2. Immunized mice produced high titers of antibodies to gC2 that both neutralized in the absence of complement and enhanced complement-mediated neutralization. Epitope mapping studies confirmed that both neutralizing and C3b binding epitopes were targeted. We then challenged mice intravaginally with a lethal dose of the HSV-2 strain MS. The gC2 mRNA-LNP (even at the lowest dose of 0.25 µg) was highly protective as a single immunogen against a lethal intravaginal HSV-2 challenge in mice. Protection correlated with antibodies that recognize distinct epitopes on gC2 involved in binding complement component C3b, highlighting the importance of targeting immune evasion domains.

## Results

### gC2 antigenic community map

**Fig 1:**
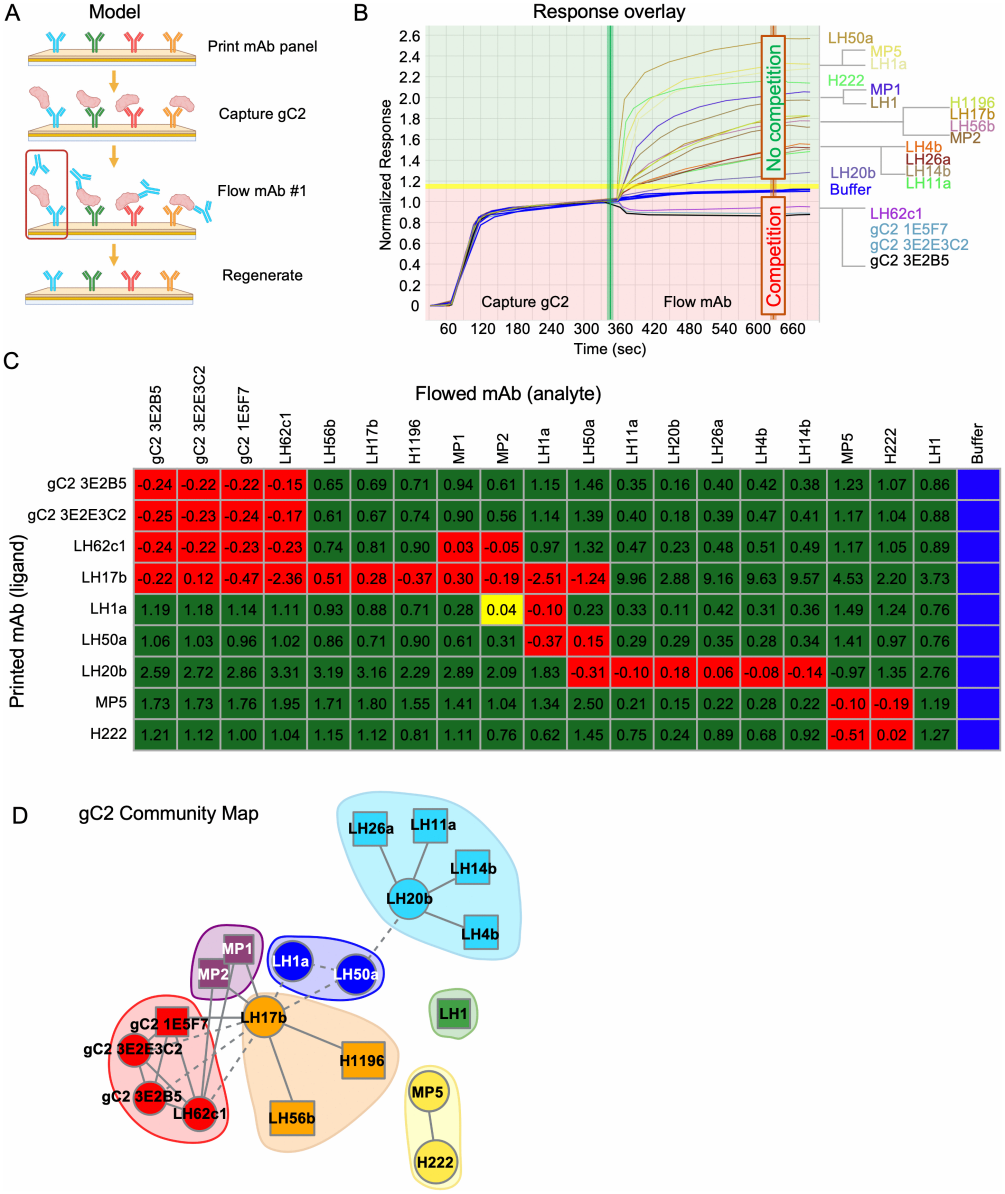
Biosensor-based antigenic community map of gC2 mAbs. **(A)** MAbs were grouped into seven communities based on competition using a biosensor-based mAb competition assay. Competition (red box) occurred when a single mAb (mAb #1) failed to bind gC2(426t) that had been captured by one or more of the printed mAbs. gC2(426t) and bound mAb were then stripped off, and the process was repeated with each additional mAb. Created in BioRender. Hook, L. (2026) https://BioRender.com/onhae59. **(B)** Example of a normalized response overlay containing all sensorgrams for repeats on one single printed mAb (gC2 3E2B5). **(C)** Heat map showing whether competition exists between the printed mAb (ligand) and the flowed mAb (analyte). **(D)** An antigenic community map was then generated for gC2.

We previously used high-throughput biosensor-based antibody competition assays to create maps of gD epitopes recognized by antibodies [24,26]. These antigenic maps group mAbs (IgG) into communities based on competition [26]. The resulting communities often contain mAbs that recognize the same or closely overlapping epitopes and that share biological function. Here, we used the high-throughput biosensor-based antibody competition assay to arrange a panel of 19 gC2 mAbs into communities (schematic, **Fig 1A and Table 1**). The 19 gC2 mAbs were obtained from a variety of sources, including our own lab, and are either type 2 specific or type common (**Table 1**). The gC2-specific mAbs were printed on a biosensor chip in discrete spots, and gC2(426t) was then captured by the printed mAbs as it flowed over the biosensor chip. A single mAb (mAb #1) was then flowed over the biosensor chip and was evaluated for binding. Competition (red box) occurred if mAb #1 failed to bind gC2(426t) that had been captured by one or more of the printed mAbs. gC2(426t) was then stripped off the printed mAbs, and the process was repeated with each additional mAb until all were evaluated. Each repeat generated a sensorgram that was normalized at the end of the gC2(426t) capture step (verticle green bar at approximately 345 seconds) (**Fig 1B**). Competition was measured near the end of the flow mAb step (verticle brown bar at approximately 630 seconds). Responses in the pink (Competition) section of the sensorgram are considered competing mAbs while responses in the green (No competition) section are considered non-competing mAbs. The thick blue curves indicate buffer controls (Buffer).

**Table 1:** Summary of the mAbs used in this study.

Name	HSV type Specificity^*#*^	Subtype	Neutralization of virus occurs in the absence of complement	Neutralization of virus is enhanced in the presence of complement	Blocks C3b binding	Community
gC2 3E2B5	2	IgG1(k)	Yes	No	No	Red
gC2 3E2E3C2	2	IgG1(k)	Yes	No	No	
gC2 1E5F7	2	IgG1(k)	Yes	No	No	
LH62c1	2	IgG2a(k)	No	No	No	
H1196	2	IgG2a(k)	Yes	Yes	Yes	Orange
LH17b	1, 2	IgG1(k)	No	Yes	Yes	
LH56b	1, 2	IgG1(k)	No	Yes	Yes	
LH1	2	IgG1(k)	No	Yes	Yes	Green
MP5	2	IgG1(k)	No	No	Yes	Yellow
H222	2	IgG2a(k)	ND	ND	Yes	
MP1	2	IgG2b(k)	No	No	Yes	Purple
MP2	2	IgG2b(k)	No	No	Yes	
LH1a	2	IgG2b(k)	No	No	No	Blue
LH50a	1, 2	IgG2a(k)	No	Yes	No	
LH4b	1, 2	IgG1(k)	No	No	No	Aqua
LH11a	1, 2	IgG1(k)	No	No	No	
LH14b	1, 2	IgG1(k)	No	ND	No	
LH20b	1, 2	IgG1(k)	No	No	Yes	
LH26a	1, 2	IgG1(k)	No	No	No	

# 2, HSV-2 type specific; 1, 2, type common; ND, not done.

Competition for all printed mAbs was then analyzed to generate a heat map (**Fig 1C**). Red boxes indicate competition, while green boxes indicate no competition. The yellow box indicates low-level binding, yet no competition. Mabs that were sensitive to regeneration conditions were excluded. The number in each box indicates the difference between the normalized response of the flowed mAb (analyte) and the buffer control (blue) at the time of measurement (brown bar, 630 seconds). A gC2 mAb community map was then generated using Carterra’s Epitope software (**Fig 1D**). The gC2-specific mAbs were divided into seven different communities distinguishable by color: red, orange, green, yellow, purple, blue, and aqua. Circles indicate that competition was measured when the mAb was evaluated as both a ligand (bound to the chip) and as an analyte (flowed over the chip); squares indicate that competition was measured in one direction only, when the mAb was evaluated as either a ligand or an analyte. Solid lines indicate that competition between the two mAbs was seen as both a ligand and an analyte for each, while dashed lines indicate that competition between mAbs was seen only in one direction [24,26].

### Association of mAb community with biological function

**Fig 2:**
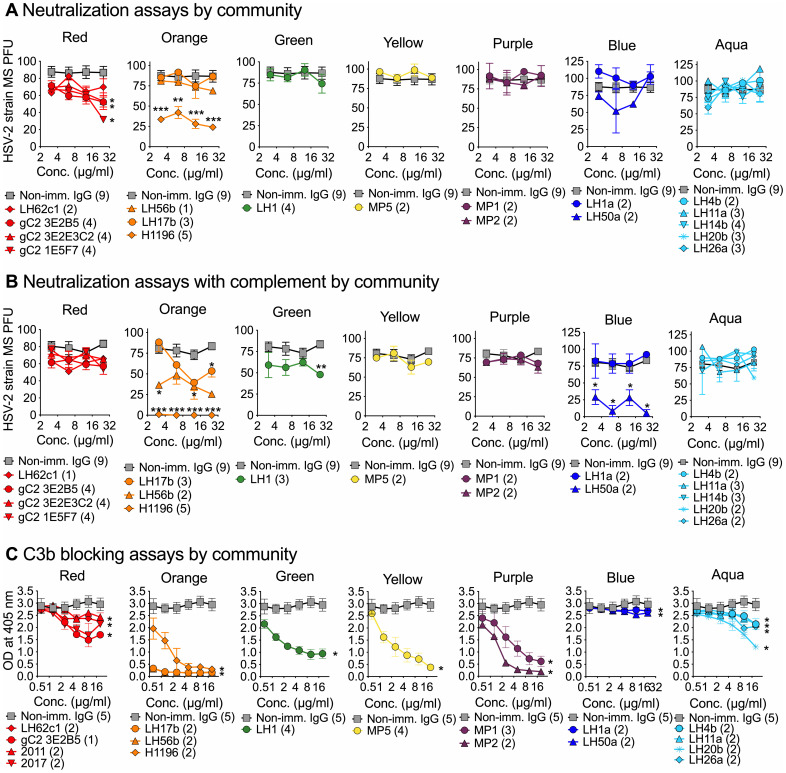
Biological functions of gC2 mAbs. **(A, B)** Neutralization of the HSV-2 strain MS by gC2 mAb in the absence of complement **(A)**, or presence of complement **(B)**, compared to Non-immune mouse IgG (Non-imm. IgG). **(C)** C3b blocking assay by gC2 mAb compared to Non-imm. IgG. Data are represented as mean ± SEM with replicate number included in brackets **(A-C)**. Statistics shown were determined by Mutiple Mann-Whitney test (**A**, **B**) and the Wilcoxon matched-pairs signed-rank test **(C)**. *, p < 0.05; **, p < 0.01; ***, p < 0.001, ****, p < 0.0001.

We evaluated the mAbs from each of the seven communities to determine which mAbs neutralize HSV-2 in the absence of complement (**Fig 2A**). MAbs gC2 3E2B5, gC2 3E2E3C2, gC2 1E5F7, and H1196 showed moderate levels of neutralizing activity (roughly 50% that of the control). The first three were grouped into the red community by the biosensor-based competition assay, which indicates that this community may recognize one or more neutralizing epitopes and can be designated as a ‘neutralizing community’. H1196 belongs to a different community (orange) and likely recognizes a different neutralizing epitope than members of the red community. None of the other gC2 mAbs neutralized the virus in the absence of complement.

We also evaluated the mAbs to determine which mAbs enhance neutralization of the virus in the presence of complement (**Fig 2B**). MAbs LH1, LH17b, LH56b, and LH50a showed moderate levels of neutralizing activity in the presence of complement (roughly 50% that of control), while H1196 showed greatly enhanced neutralizing activity. These mAbs were grouped into three different communities: LH1 (green); LH17b, LH56b, and H1196 (orange); and LH50a (blue), suggesting that three different gC2 epitopes may be involved in interacting with C3b to modulate complement.

Next, the mAbs were tested for the ability to bind gC2 and inhibit its interaction with C3b. Purified gC2(426t) was incubated with increasing concentrations of each purified mAb for 1 h and then evaluated for binding to C3b by capture ELISA (**Fig 2C**). MAbs that reduced the gC2 binding to C3b by 50% or greater compared to a control antibody were considered C3b blockers. MAbs from three different communities (orange, H1196, LH17b, and LH56b; yellow, MP5; and purple, MP1 and MP2) resulted in a significant reduction in gC2 binding to C3b (> 80% reduction compared to a control antibody). Two additional mAbs, LH1 and LH20b, from the green and aqua communities, respectively, resulted in approximately 70% and 50% reductions. Several distinct communities contain members designated as C3b blockers, indicating that multiple distinct epitopes are involved in the interaction between gC2 with C3b. Interestingly, H1196 from the orange community functions as both a neutralizing antibody and a C3b blocking antibody, suggesting some overlap among the epitopes. None of the other gC2 MAbs blocked gC2 from binding C3b.

### Vaccine-induced antibody responses in mice

#### Serum IgG ELISA.

**Fig 3:**
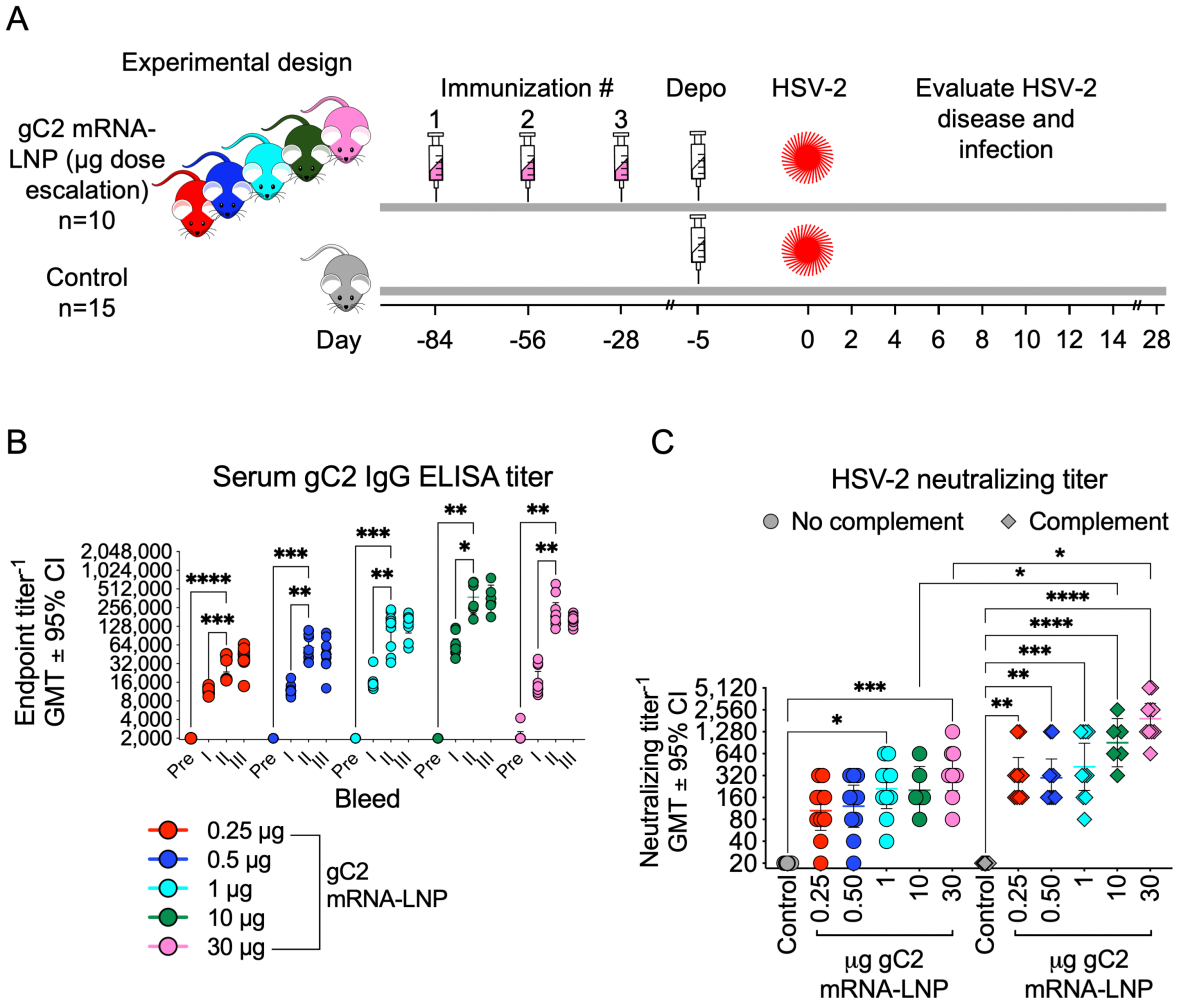
gC2 mRNA-LNP immunogenicity as a single immunogen. (**A**) Experimental design. Mice were immunized with 0.25, 0.50, 1, 10, or 30 µg gC2 mRNA-LNP i.d. 3 times, 28 days apart (n=15 for the Control group; n=10 for all gC2 vaccine groups, except for the 10 µg gC2 mRNA-LNP group, which had 6 mice at HSV-2 challenge due to 4 deaths unrelated to experimental design). Sera were obtained from mice just prior to the start of immunization and four weeks after each immunization. (**B**) Endpoint gC2 serum IgG ELISA titers. (**C**) Neutralizing antibody titers in the absence or presence of 5% HSV-1/-2 seronegative human serum as the source of complement (circles and diamonds, respectively). Data are represented as geometric mean titer  ±  95% CI (**B**-**C**). Statistics were determined by mixed-effects analysis for multiple comparisons (**B**) and Kruskal-Wallis test adjusted for multiple comparisons (**C**). *, p  <  0.05; **, p  <  0.01; ***, p  <  0.001, ****, p  <  0.0001.

The trivalent gC2, gD2, gE2 mRNA-LNP vaccine elicited high antibody titers to all three vaccine antigens and was highly protective and durable in preclinical mouse and guinea pig models [9,22]. Antibodies that correlate strongly with protection were produced to crucial gD2 epitopes involved in virus entry and cell-to-cell spread [9,23]. We extended these studies to evaluate epitope-specific responses in gC2-immunized mice. Female BALB/c mice were immunized intradermally (i.d.) 3 times at 28-day intervals with 0.25, 0.50, 1, 10, or 30 µg gC2 mRNA-LNP (**Fig 3A**).

Sera were collected just prior to the first immunization and four weeks after the first, second, and third immunizations and evaluated for IgG antibody titers to gC2 by ELISA (**Fig 3B**). Endpoint titers to gC2 generally increased with increasing dose of gC2 mRNA-LNP (0.25-10 µg) (**Fig 3B**). We detected highly significant increases in endpoint titers from doses 0.25-10 µg after the first (I) and second (II) immunization with no further increase after the third (III) (**Fig 3B**). The benefit of increased gC2 mRNA-LNP doses ended with the 10-µg dose, as a slight drop in endpoint titer was observed at 30 µg (**Fig 3B**).

#### Neutralizing antibodies in the absence and presence of complement.

HSV-2 gC2 antibodies neutralize virus in the absence of complement, possibly by interfering with the ability of gD2 to interact with other HSV-2 glycoproteins required for virus entry [27,28]. HSV-2 gC2 antibodies also block C3b binding, which enhances complement activation and renders the virus more susceptible to neutralization in the presence of complement [29–31]. To assess each of these gC2 functions, we determined the neutralizing antibody titers in the absence and presence of complement using sera collected four weeks after the third gC2 mRNA-LNP immunization (**Fig 3C**; circles and diamonds, respectively). Neutralizing antibody titers to gC2 in the absence of complement generally increased with increasing dose of the gC2 mRNA-LNP in the immunized mice (0.25-30 µg; circles) (**Fig 3C**). Neutralizing antibodies in the 1 and 30 µg gC2 mRNA-LNP groups were significantly higher than in the control group (**Fig 3C**, circles). The results suggest that gC2 mRNA-LNP induces antibodies that neutralize the virus in the absence of complement. We next evaluated neutralizing antibody titers to gC2 in the presence of 5% human complement and observed a similar positive trend between vaccine dose and neutralizing antibody titers in the gC2 mRNA-LNP immunized animals (**Fig 3C**; diamonds). Titers were significantly higher in all gC2 mRNA-LNP groups compared with the control group. The neutralizing antibody titers were also higher in the presence of complement than in the absence of complement, reaching significance for animals receiving the 10 and 30 µg doses, suggesting that gC2 antibodies are effective at activating complement, likely via the classical pathway, and perhaps some antibodies prevent gC2 from binding C3b, which further enhances complement activation.

#### Epitope-specific antibody responses.

**Fig 4:**
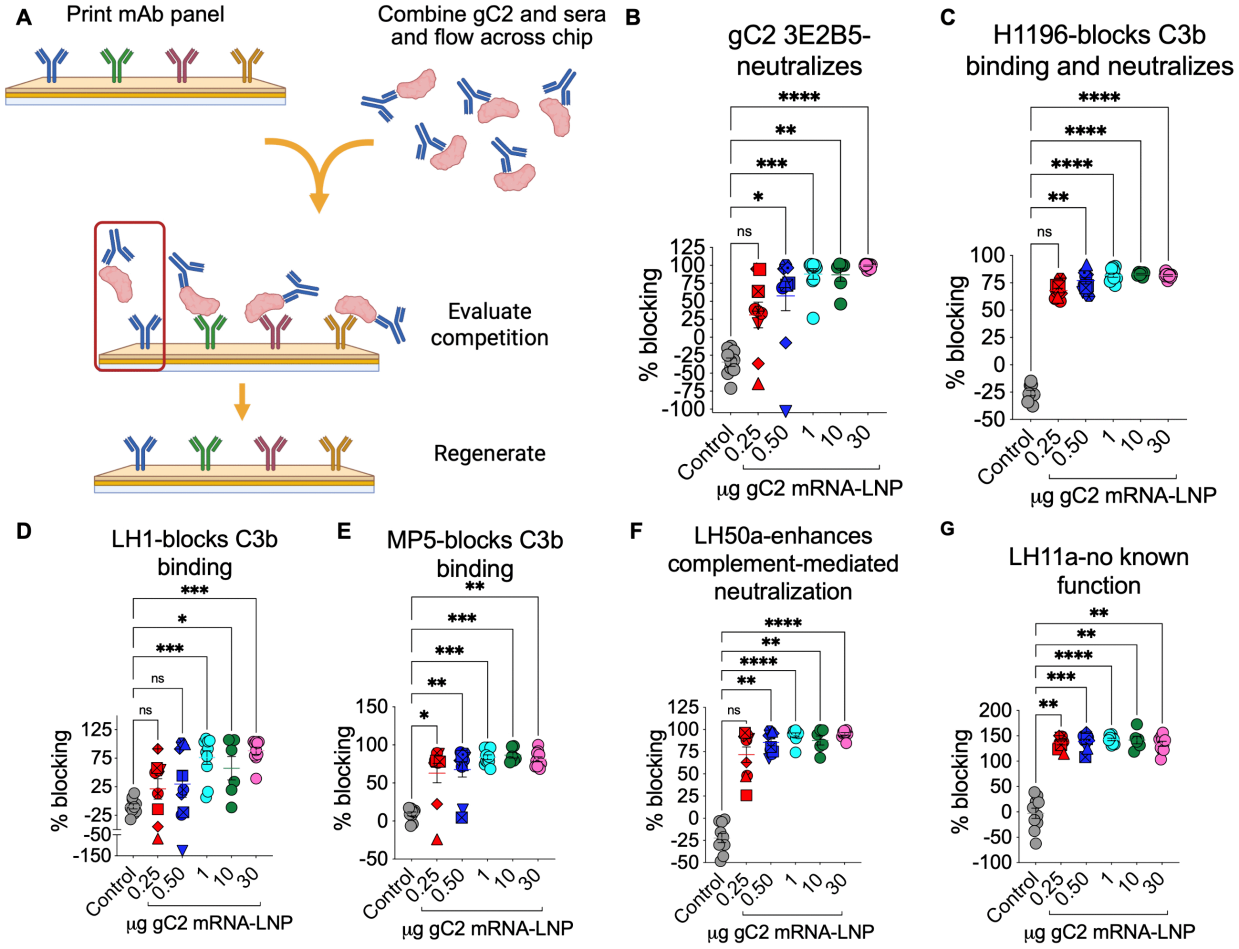
Mice immunized with gC2 mRNA-LNP produce antibodies to important functional epitopes. **(A)** Sera from immunized or control mice were evaluated for the presence of antibodies directed towards specific gC2 epitopes using a biosensor-based mAb competition assay. Competition (red box) occurred when purified gC2(426t) that had been pre-incubated with a serum sample failed to bind a gC2 mAb printed on the biosensor chip. The gC2(426t) and bound antibodies were then stripped off, and the process was repeated with each additional serum sample. Created in BioRender. Hook, L. (2026) https://BioRender.com/9pe1tw6. **(B-G)** Percent blocking was determined for each serum sample relative to a control. The shape of the symbol identifies sera from individual mice in the 0.25 and 0.5 ug gC2 mRNA dose groups; for example, the sera indicated by a diamond in (**B**) is also denoted by a diamond in **(D–G)**. Data are represented as mean ± SEM. The statistics shown were determined by the Kruskal-Wallis test, adjusted for multiple comparisons **(B-G)**. ns, not significant; *, *p* < 0.05; **, *p* < 0.01; ***, *p* < 0.001, ****, *p* < 0.0001.

We next used the high-throughput biosensor technology to define epitope-specific antibody responses to gC2 epitopes in the gC2 mRNA-LNP immunized mice [24,26]. One mAb was selected from each of the seven gC2 communities (**Fig 1D**), except for the purple community (mAbs MP1, MP2), because the mAbs in this community lost their ability to bind gC2 upon repeated regeneration cycles in the biosensor. We evaluated epitope-specific antibody responses using sera obtained 28 days after the third immunization. Serum was first incubated with purified gC2(426t) and then flowed over a biosensor chip that had been printed with multiple mAbs in discrete spots (**Fig 4A**) [24,26]. The binding of gC2 to each mAb on the chip was measured for each gC2-mouse immune serum mixture and then compared to the binding when gC2 was mixed with buffer. A reduction in gC2 binding when gC2 is mixed with serum compared to buffer indicates competition and signifies that an antibody is present in the immune serum to an epitope recognized by the mAb on the chip.

At least six sera per gC2 dose were evaluated based on availability. The red community mAb gC2 3E2B5 binds gC2 and results in HSV-2 neutralization. A majority of gC2 mRNA-LNP immunized mice generated blocking titers to gC2 3E2B5, with the 0.5, 1, 10, and 30 µg dose groups differing significantly from the control group (**Fig 4B**). The orange community mAb H1196 binds gC2, resulting in HSV-2 neutralization and also blocks gC2 from binding to complement component C3b. All gC2 mRNA-LNP immunized mice generated blocking titers to H1196, suggesting the presence of antibody in their sera to the epitope recognized by H1196, or one close by. Titers of blocking antibodies to H1196 generally increased with increasing dose of the gC2 mRNA-LNP vaccine from 0.25 to 10 µg, with no additional rise at 30 µg (**Fig 4C**). Blocking titers were significantly higher in the gC2 mRNA-LNP groups receiving the 0.5, 1, 10, and 30 µg doses than in the control group. We next evaluated the green community mAb LH1, which recognizes an epitope that is involved in binding to C3b. While blocking titers generally increased with higher doses of gC2 mRNA-LNP, we observed heterogeneity within each vaccine group (**Fig 4D**). The blocking titers at the 1, 10, and 30 µg doses, however, were significantly greater than those of the control group. We next evaluated the yellow community, mAb MP5, which also recognizes an epitope involved in C3b binding. Titers of blocking antibodies to MP5 also increased with increasing dose of the gC2 mRNA-LNP vaccine up to the 10-µg dose, with no additional increase at 30 µg (**Fig 4E**). Blocking titers were also significantly higher in the gC2 mRNA-LNP groups receiving the 1, 10, and 30 µg doses than in the control group. The blue community mAb LH50a does not prevent gC2 from interacting with complement component C3b, yet it enhances complement-mediated neutralization of the virus, suggesting it recognizes a gC2 epitope that may have novel complement-regulating properties. Mice immunized with vaccines to gC2 produce blocking titers to LH50a, and for the gC2 mRNA-LNP vaccine, appear to peak at the 1.0 µg dose (**Fig 4F**). The aqua mAb LH11a recognizes an epitope on gC2 with no known function. Blocking titers to LH11a were produced at comparable levels in all groups and differed significantly from the control group (**Fig 4G**). We conclude that mice immunized with gC2 mRNA-LNP produce antibodies to important functional gC2 epitopes involved in modulating complement activity and neutralization.

### gC2 mRNA-LNP that elicits antibodies to neutralizing and C3b binding epitopes on gC2 is highly protective

**Fig 5:**
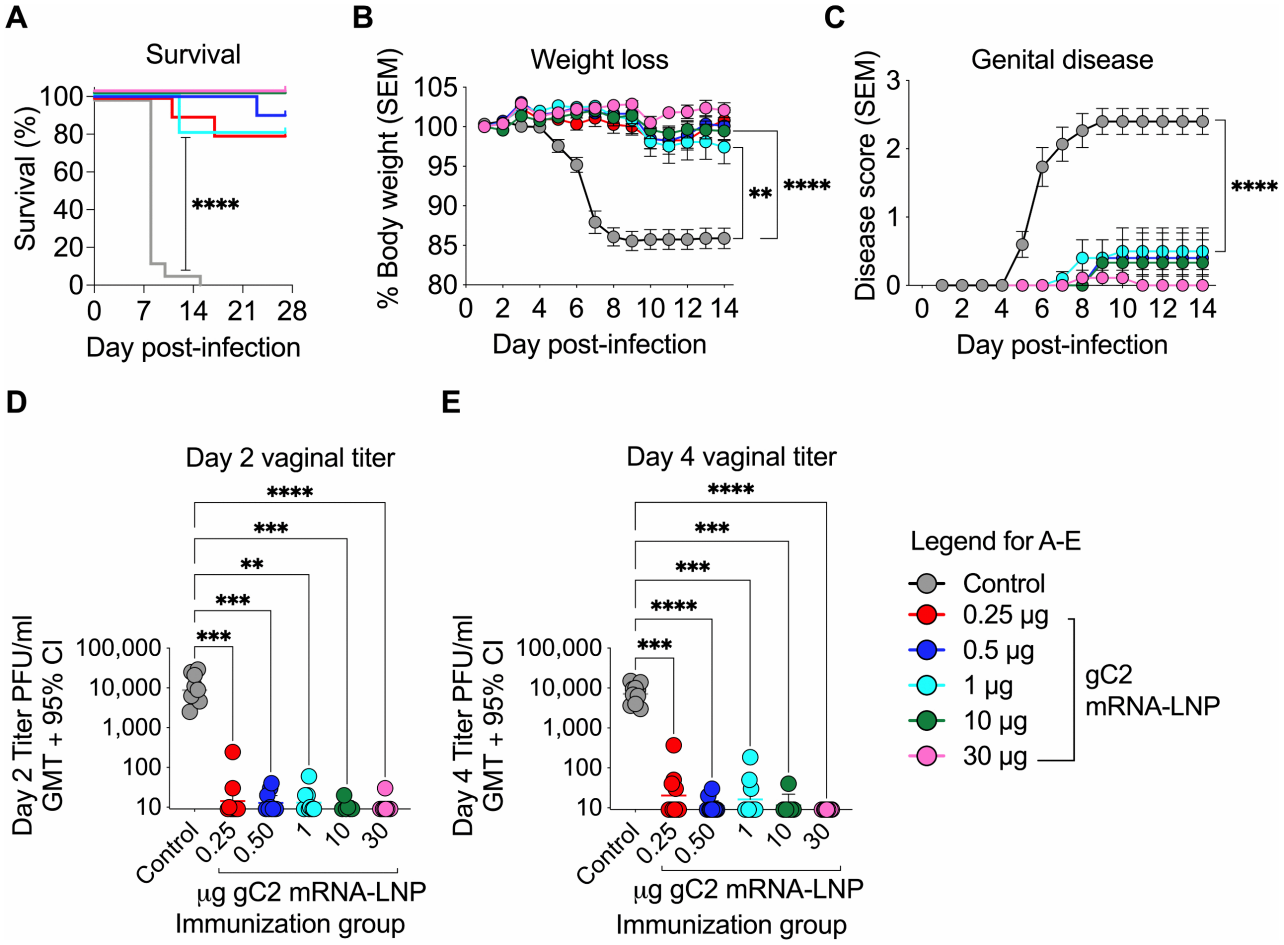
gC2 mRNA-LNP that elicits antibodies to neutralizing and C3b binding epitopes on gC2 is highly protective. Mice were immunized with 0.25, 0.50, 1, 10, or 30 µg gC2 mRNA-LNP i.d. 3 times, 28 days apart (n=15 for the Control group; n=10 for all gC2 vaccine groups, except for the 10 µg gC2 mRNA-LNP group, which had 6 mice at HSV-2 challenge due to 4 deaths unrelated to experimental design). (**A**) Survival curves. (**B**) Percent body weight post-infection. (**C**) Mean genital disease scores. (**D**, **E**) Vaginal HSV-2 virus titers on days 2 and 4 post-infection. p values in (**A**) were calculated by the log-rank (Mantel–Cox) test, in (**B**, **D**-**E**) by One-Way Anova with Dunn’s correction for multiple comparisons, and (**C**) mixed-effects analysis for multiple comparisons; *, p  <  0.05; **, p  <  0.01; ***, p  <  0.001, ****, p  <  0.0001. Depo, Depo-Provera.

Mice were challenged intravaginally 28 days after the third and final immunization with 5x10^3^ PFU HSV-2 strain MS (275 times the Lethal Dose 50% (275 LD_50_)) and evaluated for clinical disease and indicators of subclinical infection. All mice in the control group (unvaccinated) developed signs of clinical disease and required humane euthanasia by day 15, while a majority (40 of 46; 87%) of mice receiving the gC2 mRNA-LNP vaccine survived (*p* < 0.0001, Fisher’s exact test, two-tailed) (**Fig 5A**). All mice receiving the two highest doses of the mRNA vaccine (10 and 30 µg; green and pink circles respectively) survived the HSV-2 challenge. We weighed the mice during the acute phase of infection from days 1–14 post-infection (**Fig 5B**). Control mice lost significantly more weight beginning day 5 post-infection compared with each gC2 mRNA-LNP vaccine group (*p* ≤ 0.05 for all doses). No animals receiving the 10 and 30 µg doses lost more than 5% body weight.

We next evaluated mice for signs of genital disease (**Fig 5C**). Control mice developed signs of HSV-2 genital infection by day 6 post-infection, while gC2 mRNA-LNP vaccinated mice developed significantly less disease with delayed onset (~2 days later) (*p* ≤ 0.05 for each group). Few mice receiving the two highest 10 and 30 µg doses (1 of 6 and 1 of 9) showed any signs of genital disease. In summary, the gC2 mRNA-LNP vaccinated animals developed minimal disease compared with the control animals.

We next evaluated the mice for evidence of infection by measuring whether infectious virus was present in vaginal swabs obtained on days two and four post-infection (**Fig 5D and 5E**). Control animals had high titers of HSV-2 present on days two and four post-infection, while gC2 mRNA-LNP vaccinated animals had significantly lower HSV-2 titers (**Fig 5D and 5E**). By all measures, the gC2 mRNA-LNP animals consistently outperformed the control group. The results indicate that gC2 mRNA-LNP as a single immunogen is protective in mice.

#### Antibodies that target C3b binding epitopes on gC2 block gC2 from binding both human and mouse C3b.

**Fig 6:**
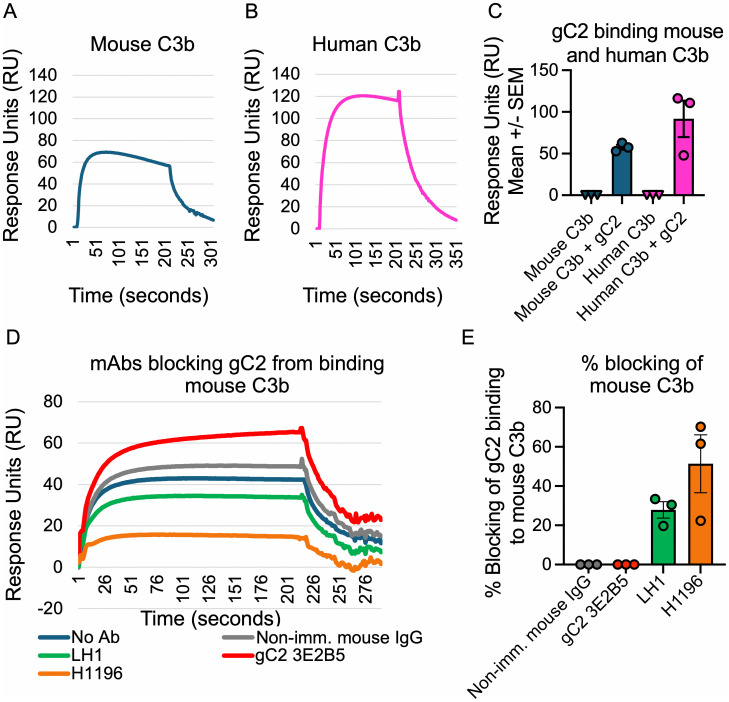
Antibodies that target C3b binding epitopes on gC2 block gC2 from binding both human and mouse C3b. **(A, B)** Representative SPRi curves; gC2 was captured by its His tag to a CM5 anti-His SPRi chip, then mouse or human C3b was flowed over the chip. The RU values demonstrate the relative amounts of mouse (**A**) or human **(B)** C3b that bound to gC2 over time. **(C)** Mean RU + /- SEM of three independent experiments evaluating mouse and human C3b binding to gC2. **(D)** Representative SPRi curves of mouse C3b binding after sequential flow of gC2; mAbs (H1196, LH1, gC2 3E2B5), non-immune mouse IgG (non-imm. mouse IgG) or buffer alone (no Ab control); followed by running buffer. The baseline RU for each response curve was established prior to the addition of mouse C3b using the running buffer and is equal to zero in the graph. **(E)** % blocking of gC2 binding to mouse C3b by mAbs H1196, LH1, gC2 3E2B5 compared with non-imm. mouse IgG. % blocking was calculated near the end of the mouse C3b injection just prior to 225 seconds using the following equation: % blocking = 100 - [(RU Test/RU no Ab)*100]. Negative values for % blocking, indicative of increased binding compared to the no Ab control, were set to zero.

Mice immunized with gC2 mRNA-LNP produce high titers of antibodies to neutralizing and C3b binding epitopes on gC2, resulting in marked protection from severe clinical disease and viral infection. We hypothesize that these antibodies neutralize HSV-2 and bind gC2 epitopes that enhance complement activation by blocking C3b. We have previously shown that murine antibodies are able to bind gC2 epitopes to block C3b isolated from human sera, but we have not determined whether murine antibodies can block C3b isolated from mouse sera [9,31]. Therefore, we designed experiments to determine whether gC2 is able to bind mouse C3b, and then evaluated whether murine antibodies are able to block mouse C3b binding (**Fig 6**) [32].

Purified gC2(445t) was captured on a CM5-anti His chip in parallel flow cells via a C-terminal His-tag present on gC2(445t). Mouse C3b was then injected across the chip to allow for possible binding in one flow cell compared to an injection of human C3b in a second flow cell (**Fig 6A and 6B**). Control flow cells included mouse or human C3b injected in the absence of gC2. Three independent experiments were performed and plotted, showing that gC2 can bind mouse C3b, albeit to a lesser extent than human C3b (**Fig 6C**) [32]. No binding of mouse or human C3b was detected in the absence of gC2 (Fig 6C).

We next evaluated whether mAbs that block gC2 from binding human C3b also block gC2 from binding mouse C3b. Three gC2-specific mAbs were selected for analysis by biosensor. These included mAb H1196 that neutralizes HSV-2 and blocks human C3b binding, mAb gC2 3E2B5 that only neutralizes, and mAb LH1 that only blocks. Purified gC2(445t) was captured on a CM5-anti His chip followed by injections of a gC2 mAb, non-immune mouse IgG or buffer alone (no Ab control) and finally mouse C3b (**Fig 6D**). Three independent experiments were performed and percent blocking of mouse C3b relative to a no Ab control was plotted (**Fig 6E**). When mAb gC2 3E2B5 that neutralizes HSV-2 was injected prior to mouse C3b, we observed an increase in RUs, indicative of increased mouse C3b binding (and no blocking), suggesting possible conformation changes in gC2 after mAb binding (**Fig 6D**). When mAbs H1196 and LH1 that block gC2 binding to human C3b binding were injected prior to mouse C3b, we observed a reduction in RUs indicative of reduced mouse C3b binding to gC2 (**Fig 6D**), which was recorded as an increase in % blocking (**Fig 6E**). Together, these data indicate that gC2 is able to bind mouse C3b, and that Abs targeting C3b binding epitopes on gC2 (whether monoclonal or polyclonal) block.

#### Antibodies that target distinct C3b binding epitopes are protective in passive transfer studies.

**Fig 7:**
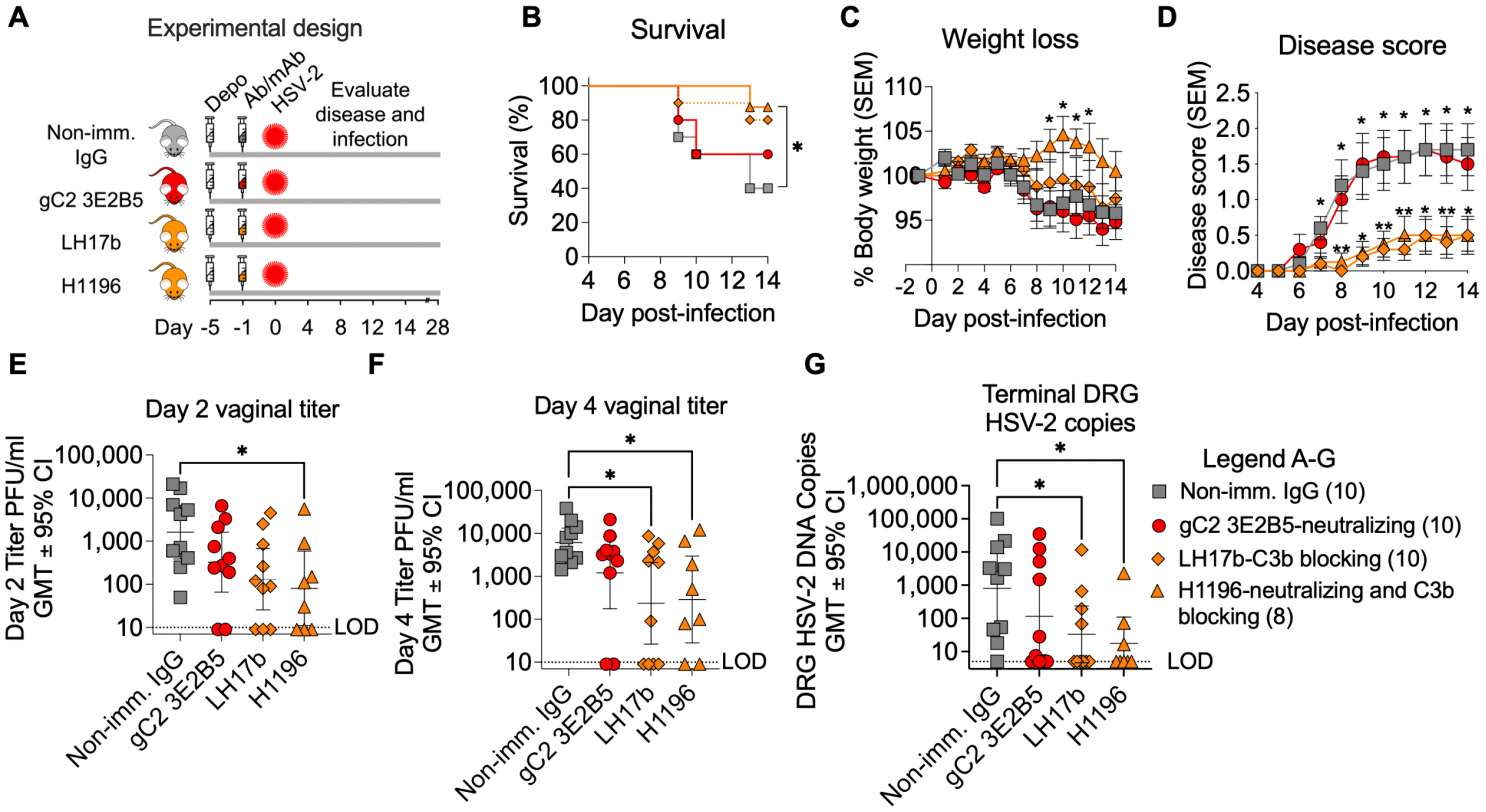
In passive transfer studies, mAb H1196 that blocks C3b binding and is neutralizing and LH17b that blocks C3b are protective (Expt 1). **(A)** Schematic of experimental design. **(B-G)** Mice were monitored for survival **(B)**, weight loss **(C)**, genital disease **(D)**, day 2 and 4 vaginal virus titers **(E, F)**, and HSV-2 DNA in DRG **(G)**. The *p*-values in (**B**) were calculated by the log-rank (Mantel–Cox) test, in (**C**–**D**) by multiple unpaired t-test with Welch’s correction for multiple comparisons, and in (**E**–**G**) by Kruskal-Wallis test for multiple comparisons. *, *p* < 0.05; **, *p* < 0.01; Depo, medroxyprogesterone.

We next evaluated whether gC2 prototype mAbs from several communities were protective in antibody passive transfer studies in mice. Our main goal was to determine whether mAbs to important gC2 epitopes *in vitro* provide protection against HSV-2 infection *in vivo*. For the first experiment (Expt. 1), we selected mAb gC2 3E2B5 that neutralizes virus, H1196 that neutralizes and blocks C3b binding, and LH17b that is closely related to H1196, but only blocks C3b binding (**Fig 7A**). Each of the three mAbs is predicted to bind a different epitope on gC2 based on their inclusion in different communities (red versus orange for gC2 3E2B5 and H1196/LH17b) and functional characteristics (neutralizing and C3b blocking versus C3b blocking only for H1196 and LH17b respectively). Non-immune murine IgG was included as a control.

Animals were inoculated i.p. with 100 μg of mAb or non-immune murine IgG as a control one day prior to intravaginal infection with an attenuated passage of HSV-2 strain MS (1x10^3^ PFU/mouse, approximately 2 LD_50_). Seven of 8 mice that received H1196 and eight out of 10 mice that received LH17b survived compared to six of 10 in the gC2 2E3B5 group, and four of 10 in the non-immune murine IgG control group (**Fig 7B**). Weight loss and genital disease followed a similar pattern, with H1196 and LH17b losing slightly less weight (**Fig 7C**). Importantly, animals passively immunized with H1196 (neutralizing and blocks C3b binding) and LH17b (blocks C3b binding) had significantly less disease than gC2 3E2B5 (neutralizing) and the control animals (**Fig 7D**). Day 4 vaginal swab titers and HSV-2 DNA copy number in dorsal root ganglia (DRG) were significantly lower in the animals that received H1196 and LH17b than in non-immune IgG animals, while differences between gC2 3E2B5 and non-immune IgG did not reach statistical significance (**Fig 7E-7G**). We conclude that a mAb that targets a neutralizing and a C3b binding epitope (H1196), or a C3b binding epitope (LH17b) protects *in vivo*. We did not detect any significant differences between the neutralizing mAb, gC2 3E2B5, and the non-immune IgG control group, although where differences appeared, they favored the neutralizing mAb (**Fig 7B and 7E-7G**).

**Fig 8:**
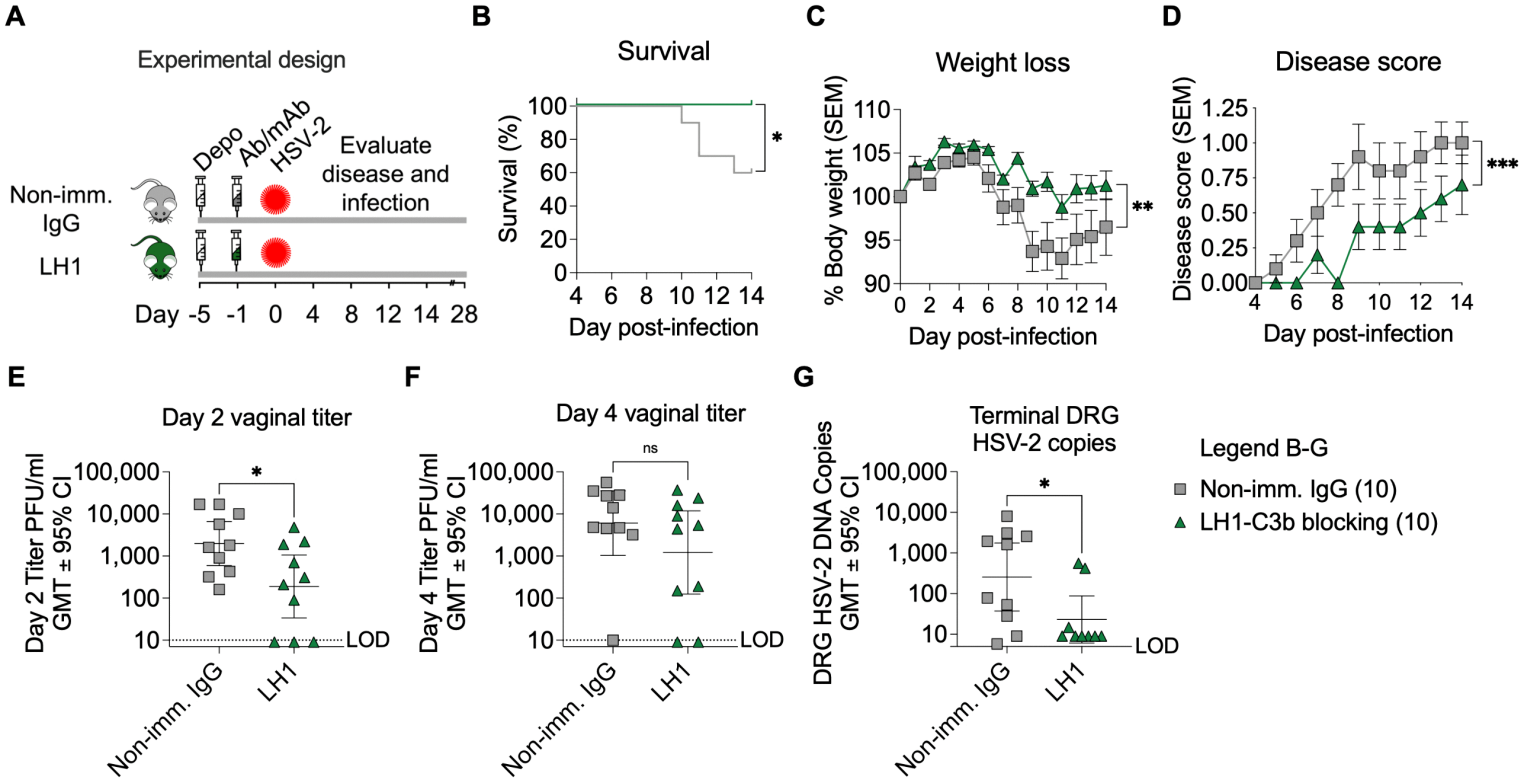
mAb LH1 that targets a C3b binding epitope is protective in passive transfer studies (Expt 2). (**A**) Schematic of experimental design. (**B**-**G**) Mice were monitored for survival (**B**), weight loss (**C**), genital disease (**D**), day 2 and 4 vaginal virus titers (**E**, **F**), and HSV-2 DNA in DRG (**G**). The p values in (**B**) were calculated by the log-rank (Mantel–Cox) test, in (**C**–**D**) by One-Way Anova with Dunn’s correction for multiple comparisons, and in (**E**–**G**) by the two-tailed Mann–Whitney test or unpaired t test, depending upon normality. *, p  <  0.05; **, p  <  0.01; ***, p  <  0.001; Depo, medroxyprogesterone.

In a second experiment (Expt. 2), we evaluated the protection provided by mAb LH1, which blocks gC2 binding to C3b but does not neutralize HSV-2. We selected LH1 because it reduces viral titer *in vitro* to the same level as gC2 3E2B5 (**Fig 2B**), but by a different mechanism. While gC2 3E2B5 neutralizes virus in the absence of complement (**Fig 2A**) by interfering with the virus’ own entry mechanism, we observed no boost in neutralization once complement was present (**Fig 2B**). In contrast, LH1 does not neutralize virus in the absence of complement (**Fig 2A**) indicating that it does not interfere with the virus’ own entry mechanism, but rather neutralizes when complement is added, possibly because LH1 blocks gC2 from binding C3b (**Fig 2B and 2C**). The experimental design for mAb LH1 (**Fig 8A**) was like that in Fig 7. Survival, weight loss, genital disease scores, day 2 vaginal swab titers, and HSV-2 DNA copy number in DRG were all significantly better in the animals that received LH1 than in the control group, with only day 4 vaginal swab titers not differing significantly between the two groups (**Fig 8B-8G**). The results strongly support that blocking C3b binding to gC2 is an immune correlate of protection, and that epitopes recognized by H1196, LH17b and LH1 are distinct and important for this activity.

## Discussion

We previously reported that the gC2/gD2/gE2 trivalent nucleoside-modified mRNA-LNP vaccine was highly effective at preventing HSV-2 genital infection in mice and guinea pigs, and that the protection provided by the trivalent mRNA vaccine was greater than the same immunogens administered as subunit proteins with CpG and alum, or when gD2 was used as a single immunogen as an mRNA-LNP vaccine [9,22]. A goal of the current study was to better understand the contribution of antibodies to gC2 to the overall protection provided by the trivalent mRNA vaccine. HSV-1 gC1 is involved in virus attachment to cells via heparan sulfate residues on the cell surface, although a similar function has not been established for gC2 [33,34]. Functions assigned to gC2 antibodies include inhibiting complement inactivation by blocking gC2 binding to C3b and neutralizing the virus, both in the presence and absence of complement [31,35]. Although antibodies to gC2 neutralize HSV-2, the potency of these antibodies is generally reduced compared to gD2 or gB2 antibodies, particularly in the absence of complement [31,36]. Our intent was to assess the contribution of gC2 immunization, in the absence of gD2 or gE2, in protecting against HSV-2 genital infection, and to evaluate mechanisms for the protection.

We immunized mice with gC2 mRNA-LNP vaccine using doses ranging from 0.25 μg to 30 μg. We demonstrate that the lowest dose of gC2 mRNA-LNP, 0.25 μg, was highly protective against a lethal intravaginal HSV-2 challenge, resulting in increased survival, less weight loss, less genital disease, and lower vaginal virus titers on days 2 and 4 post-inoculation compared to controls. We were surprised that doses as low as 0.25 ug were highly protective, resulting in few breakthrough infections and little range in disease outcomes in the gC2 mRNA-LNP immunized animals. Our vaccination scheme was designed to generate a range of immunological responses after immunization and HSV-2 disease outcomes after virus challenge with the intent of evaluating correlates of protection of the gC2 mRNA-LNP. We investigated whether there was a correlation between survival or genital disease and ELISA titers, neutralizing titers or neutralizing titers with complement, and did not find any strong correlations, likely due to the limited range of outcomes in the gC2 mRNA-LNP immunized animals. Future experiments can assess immunizing at doses < 0.25 ug gC2 to expand the range of outcomes.

We therefore evaluated ELISA and neutralizing antibody responses and HSV-2 disease outcomes comparing the mRNA and control animals. Serum IgG ELISA and neutralizing antibody titers were higher in the majority of the mRNA vaccinated animals compared with pre-vaccine sera and/or controls. Differences were most pronounced for neutralizing antibody titers in the presence of complement. As a possible explanation, the gC2 mRNA-LNP vaccine produced high titers of antibodies (% blocking) to gC2 epitopes that enhance complement activation either by blocking C3b binding (H1196, MP5, LH1) or by an unknown mechanism (LH50a). These results suggest that antibodies that interfere with the ability of gC2 to inhibit complement activation may explain the improved performance of the gC2 mRNA-LNP group compared to controls. The antibody passive transfer experiments confirmed the importance of preventing gC2 from binding C3b.

While the trivalent gC2/gD2/gE2 vaccine was initially designed as a prophylactic vaccine to prevent genital herpes caused by HSV-2, the vaccine has also shown efficacy in preclinical studies against HSV-1, including against HSV-1 genital, ocular and orolabial infection [37–39]. Homology between the three HSV-1 and HSV-2 glycoproteins exists and results in cross-reactive antibodies that prevent HSV-1 infection. gC1 and gC2 share ~65% aa sequence similarity, nearly all of which is in the carboxy-terminal four-fifths of the protein where all 8 cysteines are completely conserved and all domains responsible for C3b binding are located [40]. Only gC1 is involved in virus attachment to cells via heparan sulfate residues on the cell surface and interference with C5 and properdin binding, both attributed to its amino-terminus that is absent in gC2 [30,33].

In these studies, we focus on epitope-specific responses to gC2. We show that mice immunized with gC2 mRNA-LNP produce antibodies to five important functional epitopes recognized by the prototype mAbs gC2 3E2B5, H1196, LH1, MP5 and LH50a and a sixth epitope with no known function recognized by LH11a. Four of 6 of these mAbs (gC2 3E2B5, H1196, LH1, MP5) are type 2 specific (Table 1), suggesting that the vaccine-induced antibodies to these 4 epitopes are also type 2 specific. Interestingly, mAbs LH50a and LH11a are type common, suggesting that vaccine-induced antibodies to these epitopes may recognize gC1 and gC2 and have the potential to protect against HSV-1 and HSV-2. We are currently performing studies to map these type common and type specific epitopes on gC1 and gC2, to determine all their functions and to evaluate whether antibodies directed to them are protective. Future studies also include performing the high-throughput antibody competition assay with gC1 protein and a panel of gC1 specific mAbs to evaluate cross-reactive antibody responses to gC2 at the epitope level.

Many human pathogens encode complement-interfering molecules, including viruses, bacteria, fungi, and protozoa. The viruses include HSV-1, HSV-2, HCMV, KSHV, and vaccinia (smallpox) [35,41–45]. The bacteria include Staphylococcus aureus, Streptococcus pyogenes, Neisseria meningitides, and Borrelia burgdorferi [46–49]. The fungi and protozoa include Candida albicans, Leishmania, and Trypanosoma cruzi [50–52]. The broad expression of complement-interfering molecules by many microorganisms provides strong support for their importance in pathogenesis. Our vaccine candidate is perhaps the only one to intentionally target immune evasion molecules as part of a vaccine strategy [53]. If successful in human trials, this approach may provide new vaccine targets for other important pathogens.

This study identified a possible immune correlate of protection, namely an antibody that blocks gC2 binding to C3b. We noted that 1 μg of gC2 mRNA-LNP was sufficient to produce peak titers of antibodies that block gC2 binding to C3b for each of the mAbs evaluated, including MP5, H1196, and LH1. This result suggests that we can use antibody responses that block C3b binding to gC2 as a guide for choosing the optimal gC2 dose to include in the trivalent mRNA vaccine. We previously demonstrated immune correlates of protection of antibodies to gD2, which is another immunogen in our trivalent vaccine. The antibody correlates of protection included blocking gD2 binding to HVEM and nectin-1 receptors for HSV-2, interfering with the interaction between gD2 and HSV-2 entry glycoproteins gH2 and gL2, and blocking virus cell-to-cell spread [24]. Identifying the immune correlates of protection for each immunogen in the trivalent mRNA-LNP vaccine will help assess the quality of the antibody response in human trials.

## Methods

### Ethics statement

Female BALB/c mice were obtained from Charles River Laboratories (Wilmington, MA). The mice were used in accordance with protocol No. 805187 that was approved by the Institutional Animal Care and Use Committee of the University of Pennsylvania. The protocol follows recommendations in the Institute for Laboratory Animals Research’s “Guide for the Care and Use of Laboratory Animals.” Subcutaneous saline was given to mice that appeared dehydrated. For euthanasia, CO_2_ was administered to mice according to the recommendations of the Panel on Euthanasia of the American Veterinary Medical Association, followed by confirmation of death.

### Sex as a biological variable

Female mice were used exclusively to model protection conferred by vaccination and passive transfer of mAbs after intravaginal inoculation of HSV-2. No established genital HSV-2 infection model exists for male mice.

### Virus, gC2 protein, and mAbs

The HSV-2 strain MS used in this study was grown and titered on African green monkey kidney (Vero, ATCC) cells in 5% FBS DMEM [24,31]. Baculovirus gC2(426t) includes gC2 amino acids 27–426, from the first amino acid after the signal sequence to prior to the transmembrane domain. Baculovirus gC2(426t) was produced in insect sf9 cells and purified from the supernatant fluids as previously described [54,55]. gC2(445t) includes amino acids 28–445 (ending just prior to the transmembrane helix that starts at 448) and a 6x His tag at the C-terminus. gC2(445t) was expressed and purified from transfected CHO cells, prepared by GenScript. The gC2-specific mAbs MP1, MP2, and MP5 have been previously described [56]. MAbs H1196 and H222 were purchased from Virusys (now SeraCare). gC-specific mAb producing hybridoma cell lines gC2 3E2B5, gC2 3E2E3C2, and gC2 1E5F7 were developed by the group of Dr. Tomas Bergstrom [57]. Additional gC2 mAb producing hybridoma cell lines–LH1, LH1a, LH4b, LH11a, LH14b, LH17b, LH20b, LH26a, LH50a, LH56b, and LH62c1–were produced by the University of Pennsylvania Perelman School of Medicine Cell Center Services Core Facility (RRID:SCR_022391). Briefly, two six- to eight-week-old female BALB/c mice were immunized twice i.d., 28 days apart, with 10 µg of gC2 mRNA-LNP. Four days after the second immunization, spleen cells from the immunized mice were fused with mouse myeloma cells using polyethylene glycol. The hybrids were selected in HAT (hypoxanthine, aminopterin, and thymidine) culture medium and subcloned by limiting dilution into 10 96-well plates. Supernatant fluids from clones were collected and evaluated for HSV specificity by ELISA using gC1 and gC2 antigens baculovirus gC1(457t) and gC2(426t), respectively [54,55]. Positive clones were then subcloned and rescreened for HSV specificity. IgG subtype specificity was determined using peroxidase-conjugated class-specific anti-mouse reagents and confirmed using the IsoStrip Mouse Monoclonal Antibody Isotyping Kit according to the manufacturer’s instructions (Roche Life Science, Mannheim, Germany).

### Characterization of gC2-specific mAbs

#### gC2 neutralization assay.

A virus neutralization assay was used to evaluate mAbs to gC2 that bind gC2 and neutralize the virus in the absence or presence of 5% HSV-1 and -2 seronegative human serum as a source of complement. MAbs at the concentrations indicated were incubated with 100 PFU of HSV-2 and human complement where indicated at 37 °C for 1 h. The virus remaining after 1 h was determined by plaque assay on Vero cells. MAbs that reduced the virus plaque number by 50% or greater compared to Non-immune mouse IgG were considered neutralizers [54].

#### gC2 blocking assay.

An ELISA-based assay was used to evaluate mAbs to gC2 that blocked the interaction of gC2 with C3b [58,59]. 96-well High Binding Costar microtiter plates (Corning Incorporated, Corning, NY) were coated with purified human C3b (Complement Technologies, Inc., Tyler, TX) at 200 ng/well in 50 mM sodium bicarbonate pH 8.5-9.0 binding buffer, incubated for 1 h at room temperature and then overnight at 4°C, and blocked for 2 h at room temperature with 5% (wt/vol) nonfat milk in PBST. MAbs at the concentrations indicated were incubated with 50 ng bac-gC2(426t) for 1 h at 37°C and added to C3b-coated plates for 1 h. Bound gC2 was detected using rabbit anti-gC2 serum (UP2151) at a 1:1000 dilution, followed by a 1:2000 dilution of HRP-conjugated goat anti-rabbit IgG (GE Healthcare, Pittsburg, PA) [54]. ABTS 1-Component Microwell Peroxidase Substrate (Sera Care, Milford, MA) was added to each well and the optical density was determined at 405 nm.

#### High-throughput biosensor-based antibody competition assay.

The antibody competition assays were performed using the Carterra LSA surface plasmon resonance imaging system (Salt Lake City, UT, USA) [24,26]. MAbs that recognize gC2 were amine-coupled to an HC200M chip (Carterra). To generate the gC2 mAb community map, a competitive epitope binning assay was performed by flowing 0.1 μM soluble gC2(426t) across the chip, followed by each gC2 mAb and a regeneration solution (10 mM glycine HCl, pH 2.25) in cycles. gC2(426t) followed by buffer was flowed across the chip for the first 2–3 cycles and every 10^th^ cycle to establish the baseline compared to gC2 followed by each mAb. The data were recorded as response units (RU) and competition among mAbs was evaluated using the Epitope software and displayed as a community map (Carterra).

To define epitope-specific antibody responses to gC2 in mice, antibody competition assays were performed after amine-coupling gC2 mAbs to the HC200M chip using a pre-mix assay format by saturating 0.1 μM soluble gC2(426t) with 2% (1:50 dilution) mouse sera. Each gC2(426t)/mouse serum mix was flowed across the chip spotted with the gC2 mAbs. gC2 alone was flowed across the chip for the first 2–3 cycles and every 10th cycle to establish the background binding of gC2 compared to gC2 plus mouse serum. Data were recorded as RU. The blocking activity of mouse sera was calculated for each mAb as a percentage using the formula: [1 − (RU gC2(426t) + mouse serum)/(RU gC(426t) alone)] × 100. Occasionally, the (RU gC2(426t) + mouse serum) yielded a negative RU value that was lower than the baseline value of buffer alone, resulting in percent blocking values that exceeded 100%. Those values exceeding 100% were reported as such.

### Evaluating the interaction between gC2 and mouse C3b by biosensor

Purified gC2(445t) (~200 RU) at a concentration of 20 µg/mL was captured on a CM5-anti His chip via its C-terminal His-tag with a flow rate of 10 µl/min in HBS-EP+ (10 mM of HEPES, 150 mM NaCl, 3 mM EDTA, pH 7.4) using a Biacore 1k+ instrument (Cytiva). Mouse or human C3b (Complement Technology Inc., Tyler, TX) at a concentration of 200 µg/ml, with a flow rate of 5 µl/min in HBS-EP+ or HBS-EP+ alone, was then injected across separate flow cells for 200 sec. Controls included flow cells where only mouse, human C3b or HBS-EP+ alone were injected (no gC2).

For blocking assays, ~ 200 RU of purified gC2(445t) was first captured on a CM5-anti His chip as above. Murine non-immune IgG (IgG from mouse serum I5381, Sigma Aldrich) and mAbs H1196, gC2 3E2B5, and LH1 at concentrations of 40 µg/ml, flow rate 20 µl/min in HBS-EP+ or HBS-EP+ alone were then injected across separate flow cells for 60 sec. Purified mouse C3b at a concentration of 200 µg/ml, flow rate 5 µl/min in HBS-EP + was then injected over all flow cells for 225 sec. The RU from a control flow cell where only mouse C3b was injected (no gC2, no Abs/mAbs) was subtracted from each curve. The baseline RU for each response curve was established prior to the addition of mouse C3b using running buffer and is equal to zero in the graph. The % blocking of each antibody was calculated near the end of the mouse C3b injection (just prior to 225 sec) relative to the no Ab control using the following equation: % blocking = 100 - [(RU Test/RU no Ab)*100]. Negative values for % blocking, indicative of increased binding compared to the no Ab control, were set to zero.

#### Mice immunization and challenge.

Six-to-eight-week-old female BALB/c mice were purchased from Charles River Laboratories, Wilmington, MA. Mice were immunized i.d. 3 times at 28-day intervals with 0.25, 0.50, 1, 10, or 30 µg of gC2 mRNA-LNP. The gC2 mRNA-LNP used for the immunizations has been described previously [9,60]. Briefly, nucleoside-modified mRNA encoding gC2 was produced with T7 RNA polymerase (Megascript, Ambion) using m1Ψ-5′-triphosphate (TriLink) instead of UTP, capped using the m7G capping kit with 2′-*O*-methyltransferase (ScriptCap, CellScript), and purified by High-Performance Liquid Chromatography (HPLC) (Akta Purifier GE Healthcare) [61]. HPLC-nucleoside-modified mRNA was assessed for integrity on an agarose gel, encapsulated in LNPs (Acuitas), and stored at -80°C at a concentration of 1 mg/ml. The gC2 mRNA-LNP was resuspended in sterile saline at the concentrations indicated just prior to immunization. Immunizations were administered at four sites (7.5 μl/site) on the shaved flank using a 29-gauge insulin syringe.

Five days prior to challenge, mice were injected subcutaneously with 2 mg of medroxyprogesterone (Depo) to increase susceptibility to intravaginal HSV-2 infection [62]. On the day of challenge, the vagina of each mouse was cleared using a sterile cotton swab moistened with PBS, followed by an intravaginal challenge with HSV-2 MS (5X10^3^ PFU/mouse, approximately 275 LD_50_). The virus inoculum was titered after challenge to ensure an accurate dosage of 5X10^3^ PFU/mouse. Mice were evaluated 28 days post-infection for survival, weight loss, genital disease, and day 2 and day 4 vaginal viral titers. Weight was recorded daily with final weight carried forward for animals requiring euthanasia. Genital disease was scored on a scale of 0–4, with one point given each for erythema, exudate, hair loss, and necrosis of genital tissues [54]. The final disease score was carried forward for animals requiring euthanasia. Day 2 and day 4 vaginal viral titers were determined by gently swabbing the vagina with a sterile polyester swab that was then placed into a one ml volume of Dulbecco’s modified essential media supplemented with 5% fetal bovine serum (5% FBS DMEM) and 25μg/ml vancomycin. Titers were then determined by standard viral plaque assay on Vero cells [54].

#### Mice passive transfer and challenge.

Studies were performed as above with the following modifications. Mice received a passive transfer of 100 μg mAb/mouse intraperitoneally (i.p.) one day prior to an intravaginal challenge of an attenuated passage of HSV-2 MS (1x10^3^ PFU/mouse, approximately 2 LD_50_). The vagina of each mouse was not cleared with a cotton swab prior to infection to prevent removal of the passively transferred mAb.

#### ELISA endpoint titers to gC2 from sera.

ELISA endpoint titers to gC2 were determined for sera samples from each mouse. Sera were collected just prior to the start and four weeks after each immunization. To determine endpoint titers, 96-well High Binding Costar microtiter plates (Corning Incorporated, Corning, NY) were coated with purified bac-gC2(426t) at 100 ng/well in 50 mM sodium carbonate pH 8.5-9.0 binding buffer, incubated for 1 h at room temperature then overnight at 4°C, and blocked for 1 h with 5% (wt/vol) nonfat milk in PBS 0.05% Tween 20 (PBST) [54,58,59]. Plates were washed 3 times with PBST, and serial 2-fold dilutions of mouse sera in PBST beginning at a dilution of 1:2000 were added for 1 h at room temperature with orbital shaking. Plates were washed, and bound IgG was detected at an optical density of 405 nm using horseradish peroxidase-conjugated anti-mouse IgG (GE Healthcare, Pittsburgh, PA) at a 1:2000 dilution, followed by ABTS 1-Component Microwell Peroxidase Substrate (Sera Care, Milford, MA). Endpoint titers were calculated by linear regression as the IgG dilution giving an OD reading > 2-fold higher than background.

#### Neutralizing antibody endpoint titers.

Sera were incubated at 56^o^C for 30 minutes to inactivate complement present in each sample. Sera were then serially diluted in 5% FBS DMEM beginning at a dilution of 1:10 and incubated with 100 plaque-forming units (PFU) of HSV-2 strain MS for 1 h in the absence or presence of 5% HSV-1 and -2 seronegative human sera as the source of complement. The number of PFUs remaining following incubation was determined by standard plaque assay on Vero cells [31]. Neutralizing antibody titers were reported as the serum dilution that reduced the number of virus plaques by 50%.

### qPCR for HSV-2 DNA Present in the DRG

Purified DNA was isolated and evaluated by qPCR as previously described using the following primer and probe sets: HSV-2 Us9 Forward, GGCAGAAGCCTACTACTCGGAAAA; Reverse, CCATGCGCACGAGGAAA; and Probe, FAM-CGAGGCCGCCAAC-MGBNFQ and mouse adipsin: Forward, GCAGTCGAAGGTGTGGTTACG; Reverse, GGTATAGACGCCCGGCTTTT; and Probe, FAM-CTGTGGCAATGGC-MGBNFQ. DRG HSV-2 DNA copy number was expressed as log_10_ DNA copies per 10^5^ adipsin genes [9].

#### Statistical analysis.

All statistical analyses described in this study were performed using GraphPad Prism version X for macOS, GraphPad Software, San Diego, CA, www.graphpad.com. The log-rank (Mantel-Cox) test was used to calculate *p* values for survival. The D’Agostino & Pearson normality test was performed to evaluate the normality of the data. Mann-Whitney tests were used to determine statistical significance when data were not normal. One or Two-Way ANOVA tests were used as indicated in figure legends and were adjusted for multiple comparisons. Fisher’s exact test was used to evaluate proportional differences between variables. Results were considered significant at a *p* value <0.05.

## References

[1] Wald A, Link K. Risk of human immunodeficiency virus infection in herpes simplex virus type 2-seropositive persons: a meta-analysis. J Infect Dis. 2002;185(1):45–52. doi: 10.1086/338231 11756980

[2] Corey L, Adams HG, Brown ZA, Holmes KK. Genital herpes simplex virus infections: clinical manifestations, course, and complications. Ann Intern Med. 1983;98(6):958–72. doi: 10.7326/0003-4819-98-6-958 6344712

[3] Corey L, Wald A, Patel R, Sacks SL, Tyring SK, Warren T, et al. Once-daily valacyclovir to reduce the risk of transmission of genital herpes. N Engl J Med. 2004;350(1):11–20. doi: 10.1056/NEJMoa035144 14702423

[4] Kimberlin DW, Whitley RJ, Wan W, Powell DA, Storch G, Ahmed A, et al. Oral acyclovir suppression and neurodevelopment after neonatal herpes. N Engl J Med. 2011;365(14):1284–92. doi: 10.1056/NEJMoa1003509 21991950 PMC3250992

[5] Corey L, Langenberg AG, Ashley R, Sekulovich RE, Izu AE, Douglas JM Jr, et al. Recombinant glycoprotein vaccine for the prevention of genital HSV-2 infection: two randomized controlled trials. Chiron HSV Vaccine Study Group. JAMA. 1999;282(4):331–40. doi: 10.1001/jama.282.4.331 10432030

[6] Stanberry LR, Spruance SL, Cunningham AL, Bernstein DI, Mindel A, Sacks S, et al. Glycoprotein-D-adjuvant vaccine to prevent genital herpes. N Engl J Med. 2002;347(21):1652–61. doi: 10.1056/NEJMoa011915 12444179

[7] Belshe RB, Leone PA, Bernstein DI, Wald A, Levin MJ, Stapleton JT, et al. Efficacy results of a trial of a herpes simplex vaccine. N Engl J Med. 2012;366(1):34–43. doi: 10.1056/NEJMoa1103151 22216840 PMC3287348

[8] Plotkin SA. Correlates of protection induced by vaccination. Clin Vaccine Immunol. 2010;17(7):1055–65. doi: 10.1128/CVI.00131-10 20463105 PMC2897268

[9] Awasthi S, Hook LM, Pardi N, Wang F, Myles A, Cancro MP, et al. Nucleoside-modified mRNA encoding HSV-2 glycoproteins C, D, and E prevents clinical and subclinical genital herpes. Sci Immunol. 2019;4(39):eaaw7083. doi: 10.1126/sciimmunol.aaw7083 31541030 PMC6822172

[10] Yeager AS, Arvin AM, Urbani LJ, Kemp JA 3rd. Relationship of antibody to outcome in neonatal herpes simplex virus infections. Infect Immun. 1980;29(2):532–8. doi: 10.1128/iai.29.2.532-538.1980 7216423 PMC551151

[11] Brown ZA, Benedetti J, Ashley R, Burchett S, Selke S, Berry S, et al. Neonatal herpes simplex virus infection in relation to asymptomatic maternal infection at the time of labor. N Engl J Med. 1991;324(18):1247–52. doi: 10.1056/NEJM199105023241804 1849612

[12] Langenberg AGM, Corey L, Ashley RL, Leong WP, Straus SE. A Prospective Study of New Infections with Herpes Simplex Virus Type 1 and Type 2. N Engl J Med. 1999;341(19):1432–8. doi: 10.1056/nejm199911043411904 10547406

[13] Belshe RB, Heineman TC, Bernstein DI, Bellamy AR, Ewell M, van der Most R, et al. Correlate of immune protection against HSV-1 genital disease in vaccinated women. J Infect Dis. 2014;209(6):828–36. doi: 10.1093/infdis/jit651 24285844 PMC3935479

[14] Parr EL, Parr MB. Immunoglobulin G is the main protective antibody in mouse vaginal secretions after vaginal immunization with attenuated herpes simplex virus type 2. J Virol. 1997;71(11):8109–15. doi: 10.1128/JVI.71.11.8109-8115.1997 9343160 PMC192266

[15] Kohl S, Charlebois ED, Sigouroudinia M, Goldbeck C, Hartog K, Sekulovich RE, et al. Limited antibody-dependent cellular cytotoxicity antibody response induced by a herpes simplex virus type 2 subunit vaccine. J Infect Dis. 2000;181(1):335–9. doi: 10.1086/315208 10608784

[16] Eisenberg RJ, Ponce de Leon M, Friedman HM, Fries LF, Frank MM, Hastings JC, et al. Complement component C3b binds directly to purified glycoprotein C of herpes simplex virus types 1 and 2. Microb Pathog. 1987;3(6):423–35. doi: 10.1016/0882-4010(87)90012-x 2849025

[17] Hook LM, Lubinski JM, Jiang M, Pangburn MK, Friedman HM. Herpes simplex virus type 1 and 2 glycoprotein C prevents complement-mediated neutralization induced by natural immunoglobulin M antibody. J Virol. 2006;80(8):4038–46. doi: 10.1128/JVI.80.8.4038-4046.2006 16571820 PMC1440426

[18] Awasthi S, Huang J, Shaw C, Friedman HM. Blocking herpes simplex virus 2 glycoprotein E immune evasion as an approach to enhance efficacy of a trivalent subunit antigen vaccine for genital herpes. J Virol. 2014;88(15):8421–32. doi: 10.1128/JVI.01130-14 24829358 PMC4135967

[19] Galli JD, Horton M, Durr E, Heidecker GJ, Freed D, Fridman A, et al. Evaluation of HSV-2 gE Binding to IgG-Fc and Application for Vaccine Development. Vaccines (Basel). 2022;10(2):184. doi: 10.3390/vaccines10020184 35214644 PMC8879737

[20] Zhu C, Dukhovlinova E, Council O, Ping L, Faison EM, Prabhu SS, et al. Rationally designed carbohydrate-occluded epitopes elicit HIV-1 Env-specific antibodies. Nat Commun. 2019;10(1):948. doi: 10.1038/s41467-019-08876-w 30814513 PMC6393580

[21] Lin W-S, Chen I-C, Chen H-C, Lee Y-C, Wu S-C. Glycan Masking of Epitopes in the NTD and RBD of the Spike Protein Elicits Broadly Neutralizing Antibodies Against SARS-CoV-2 Variants. Front Immunol. 2021;12:795741. doi: 10.3389/fimmu.2021.795741 34925381 PMC8674692

[22] Awasthi S, Knox JJ, Desmond A, Alameh M-G, Gaudette BT, Lubinski JM, et al. Trivalent nucleoside-modified mRNA vaccine yields durable memory B cell protection against genital herpes in preclinical models. J Clin Invest. 2021;131(23):e152310. doi: 10.1172/JCI152310 34618692 PMC8631595

[23] Hook LM, Awasthi S, Cairns TM, Alameh M-G, Fowler BT, Egan KP, et al. Antibodies to Crucial Epitopes on HSV-2 Glycoprotein D as a Guide to Dosing an mRNA Genital Herpes Vaccine. Viruses. 2022;14(3):540. doi: 10.3390/v14030540 35336946 PMC8953786

[24] Hook LM, Cairns TM, Awasthi S, Brooks BD, Ditto NT, Eisenberg RJ, et al. Vaccine-induced antibodies to herpes simplex virus glycoprotein D epitopes involved in virus entry and cell-to-cell spread correlate with protection against genital disease in guinea pigs. PLoS Pathog. 2018;14(5):e1007095. doi: 10.1371/journal.ppat.1007095 29791513 PMC5988323

[25] Eisenberg RJ, Atanasiu D, Cairns TM, Gallagher JR, Krummenacher C, Cohen GH. Herpes virus fusion and entry: a story with many characters. Viruses. 2012;4(5):800–32. doi: 10.3390/v4050800 22754650 PMC3386629

[26] Cairns TM, Ditto NT, Lou H, Brooks BD, Atanasiu D, Eisenberg RJ, et al. Global sensing of the antigenic structure of herpes simplex virus gD using high-throughput array-based SPR imaging. PLoS Pathog. 2017;13(6):e1006430. doi: 10.1371/journal.ppat.1006430 28614387 PMC5484518

[27] Hull MA, Pritchard SM, Nicola AV. Herpes simplex virus 1 envelope glycoprotein C shields glycoprotein D to protect virions from entry-blocking antibodies. J Virol. 2025;99(4):e0009025. doi: 10.1128/jvi.00090-25 40135897 PMC11998518

[28] Hook LM, Huang J, Jiang M, Hodinka R, Friedman HM. Blocking antibody access to neutralizing domains on glycoproteins involved in entry as a novel mechanism of immune evasion by herpes simplex virus type 1 glycoproteins C and E. J Virol. 2008;82(14):6935–41. doi: 10.1128/JVI.02599-07 18480440 PMC2446985

[29] Fries LF, Friedman HM, Cohen GH, Eisenberg RJ, Hammer CH, Frank MM. Glycoprotein C of herpes simplex virus 1 is an inhibitor of the complement cascade. J Immunol. 1986;137(5):1636–41. doi: 10.4049/jimmunol.137.5.1636 3018078

[30] Kostavasili I, Sahu A, Friedman HM, Eisenberg RJ, Cohen GH, Lambris JD. Mechanism of complement inactivation by glycoprotein C of herpes simplex virus. J Immunol. 1997;158(4):1763–71. doi: 10.4049/jimmunol.158.4.1763 9029114

[31] Awasthi S, Lubinski JM, Shaw CE, Barrett SM, Cai M, Wang F, et al. Immunization with a vaccine combining herpes simplex virus 2 (HSV-2) glycoprotein C (gC) and gD subunits improves the protection of dorsal root ganglia in mice and reduces the frequency of recurrent vaginal shedding of HSV-2 DNA in guinea pigs compared to immunization with gD alone. J Virol. 2011;85(20):10472–86. doi: 10.1128/JVI.00849-11 21813597 PMC3187515

[32] Huemer HP, Larcher C, van Drunen Littel-van den Hurk S, Babiuk LA. Species selective interaction of Alphaherpesvirinae with the “unspecific” immune system of the host. Arch Virol. 1993;130(3–4):353–64. doi: 10.1007/BF01309666 8390825

[33] Herold BC, WuDunn D, Soltys N, Spear PG. Glycoprotein C of herpes simplex virus type 1 plays a principal role in the adsorption of virus to cells and in infectivity. J Virol. 1991;65(3):1090–8. doi: 10.1128/JVI.65.3.1090-1098.1991 1847438 PMC239874

[34] Gerber SI, Belval BJ, Herold BC. Differences in the role of glycoprotein C of HSV-1 and HSV-2 in viral binding may contribute to serotype differences in cell tropism. Virology. 1995;214(1):29–39. doi: 10.1006/viro.1995.9957 8525631

[35] Friedman HM, Cohen GH, Eisenberg RJ, Seidel CA, Cines DB. Glycoprotein C of herpes simplex virus 1 acts as a receptor for the C3b complement component on infected cells. Nature. 1984;309(5969):633–5. doi: 10.1038/309633a0 6328323

[36] Cairns TM, Fontana J, Huang Z-Y, Whitbeck JC, Atanasiu D, Rao S, et al. Mechanism of neutralization of herpes simplex virus by antibodies directed at the fusion domain of glycoprotein B. J Virol. 2014;88(5):2677–89. doi: 10.1128/JVI.03200-13 24352457 PMC3958082

[37] Egan KP, Hook LM, Naughton A, Pardi N, Awasthi S, Cohen GH, et al. An HSV-2 nucleoside-modified mRNA genital herpes vaccine containing glycoproteins gC, gD, and gE protects mice against HSV-1 genital lesions and latent infection. PLoS Pathog. 2020;16(7):e1008795. doi: 10.1371/journal.ppat.1008795 32716975 PMC7410331

[38] Egan KP, Awasthi S, Tebaldi G, Hook LM, Naughton AM, Fowler BT, et al. A Trivalent HSV-2 gC2, gD2, gE2 Nucleoside-Modified mRNA-LNP Vaccine Provides Outstanding Protection in Mice against Genital and Non-Genital HSV-1 Infection, Comparable to the Same Antigens Derived from HSV-1. Viruses. 2023;15(7):1483. doi: 10.3390/v15071483 37515169 PMC10384700

[39] Chalmin Katz A, Egan KP, Syeda Z, Son S, Watson B, Gopalakrishnan M, et al. Immunogenicity and Efficacy of a Trivalent HSV-2 gC2, gD2, gE2 Nucleoside-Modified mRNA-LNP Vaccine Against HSV-1 Eye Infection and Neuroinvasion in Mice. Vaccines (Basel). 2026;14(3):253. doi: 10.3390/vaccines14030253 41893790 PMC13030542

[40] Rux AH, Moore WT, Lambris JD, Abrams WR, Peng C, Friedman HM, et al. Disulfide bond structure determination and biochemical analysis of glycoprotein C from herpes simplex virus. J Virol. 1996;70(8):5455–65. doi: 10.1128/JVI.70.8.5455-5465.1996 8764057 PMC190503

[41] MacCormac LP, Grundy JE. Human cytomegalovirus induces an Fc gamma receptor (Fc gammaR) in endothelial cells and fibroblasts that is distinct from the human cellular Fc gammaRs. J Infect Dis. 1996;174(6):1151–61. doi: 10.1093/infdis/174.6.1151 8940203

[42] Vezzani G, Pimazzoni S, Ferranti R, Calò S, Monda G, Amendola D, et al. Human immunoglobulins are transported to HCMV viral envelope by viral Fc gamma receptors-dependent and independent mechanisms. Front Microbiol. 2023;13:1106401. doi: 10.3389/fmicb.2022.1106401 36726564 PMC9885202

[43] Rux AH, Lou H, Lambris JD, Friedman HM, Eisenberg RJ, Cohen GH. Kinetic analysis of glycoprotein C of herpes simplex virus types 1 and 2 binding to heparin, heparan sulfate, and complement component C3b. Virology. 2002;294(2):324–32. doi: 10.1006/viro.2001.1326 12009874

[44] Isaacs SN, Kotwal GJ, Moss B. Vaccinia virus complement-control protein prevents antibody-dependent complement-enhanced neutralization of infectivity and contributes to virulence. Proc Natl Acad Sci U S A. 1992;89(2):628–32. doi: 10.1073/pnas.89.2.628 1731333 PMC48292

[45] Gautam AK, Panse Y, Ghosh P, Reza MJ, Mullick J, Sahu A. Mutational analysis of Kaposica reveals that bridging of MG2 and CUB domains of target protein is crucial for the cofactor activity of RCA proteins. Proc Natl Acad Sci U S A. 2015;112(41):12794–9. doi: 10.1073/pnas.1506449112 26420870 PMC4611655

[46] Rooijakkers SHM, Ruyken M, Roos A, Daha MR, Presanis JS, Sim RB, et al. Immune evasion by a staphylococcal complement inhibitor that acts on C3 convertases. Nat Immunol. 2005;6(9):920–7. doi: 10.1038/ni1235 16086019

[47] Horstmann RD, Sievertsen HJ, Knobloch J, Fischetti VA. Antiphagocytic activity of streptococcal M protein: selective binding of complement control protein factor H. Proc Natl Acad Sci U S A. 1988;85(5):1657–61. doi: 10.1073/pnas.85.5.1657 2964038 PMC279833

[48] McNeil LK, Zagursky RJ, Lin SL, Murphy E, Zlotnick GW, Hoiseth SK, et al. Role of factor H binding protein in Neisseria meningitidis virulence and its potential as a vaccine candidate to broadly protect against meningococcal disease. Microbiol Mol Biol Rev. 2013;77(2):234–52. doi: 10.1128/MMBR.00056-12 23699256 PMC3668674

[49] Kraiczy P, Stevenson B. Complement regulator-acquiring surface proteins of Borrelia burgdorferi: Structure, function and regulation of gene expression. Ticks Tick Borne Dis. 2013;4(1–2):26–34. doi: 10.1016/j.ttbdis.2012.10.039 23219363 PMC3610323

[50] Harpf V, Rambach G, Würzner R, Lass-Flörl C, Speth C. Candida and Complement: New Aspects in an Old Battle. Front Immunol. 2020;11:1471. doi: 10.3389/fimmu.2020.01471 32765510 PMC7381207

[51] Filho AAP, Nascimento AAS, Saab NAA, Fugiwara RT, D’Ávila Pessoa GC, Koerich LB, et al. Evasion of the complement system by Leishmania through the uptake of factor H, a complement regulatory protein. Acta Trop. 2021;224:106152. doi: 10.1016/j.actatropica.2021.106152 34599886

[52] Ferreira V, Valck C, Sánchez G, Gingras A, Tzima S, Molina MC, et al. The classical activation pathway of the human complement system is specifically inhibited by calreticulin from Trypanosoma cruzi. J Immunol. 2004;172(5):3042–50. doi: 10.4049/jimmunol.172.5.3042 14978109

[53] Friedman HM. Immunologic strategies for herpes vaccination. JAMA. 2000;283(6):746; author reply 746-7. doi: 10.1001/jama.283.6.746 10683051

[54] Awasthi S, Balliet JW, Flynn JA, Lubinski JM, Shaw CE, DiStefano DJ, et al. Protection provided by a herpes simplex virus 2 (HSV-2) glycoprotein C and D subunit antigen vaccine against genital HSV-2 infection in HSV-1-seropositive guinea pigs. J Virol. 2014;88(4):2000–10. doi: 10.1128/JVI.03163-13 24284325 PMC3911559

[55] Tal-Singer R, Peng C, Ponce De Leon M, Abrams WR, Banfield BW, Tufaro F, et al. Interaction of herpes simplex virus glycoprotein gC with mammalian cell surface molecules. J Virol. 1995;69(7):4471–83. doi: 10.1128/JVI.69.7.4471-4483.1995 7769707 PMC189189

[56] Seidel-Dugan C, Ponce de Leon M, Friedman HM, Eisenberg RJ, Cohen GH. Identification of C3b-binding regions on herpes simplex virus type 2 glycoprotein C. J Virol. 1990;64(5):1897–906. doi: 10.1128/JVI.64.5.1897-1906.1990 2157859 PMC249343

[57] Adamiak B, Ekblad M, Bergström T, Ferro V, Trybala E. Herpes simplex virus type 2 glycoprotein G is targeted by the sulfated oligo- and polysaccharide inhibitors of virus attachment to cells. J Virol. 2007;81(24):13424–34. doi: 10.1128/JVI.01528-07 17928351 PMC2168860

[58] Awasthi S, Hook LM, Shaw CE, Pahar B, Stagray JA, Liu D, et al. An HSV-2 Trivalent Vaccine Is Immunogenic in Rhesus Macaques and Highly Efficacious in Guinea Pigs. PLoS Pathog. 2017;13(1):e1006141. doi: 10.1371/journal.ppat.1006141 28103319 PMC5245903

[59] Hook LM, Awasthi S, Dubin J, Flechtner J, Long D, Friedman HM. A trivalent gC2/gD2/gE2 vaccine for herpes simplex virus generates antibody responses that block immune evasion domains on gC2 better than natural infection. Vaccine. 2019;37(4):664–9. doi: 10.1016/j.vaccine.2018.11.076 30551986 PMC6447314

[60] Egan K, Hook LM, Naughton A, Friedman HM, Awasthi S. Herpes simplex virus type 2 trivalent protein vaccine containing glycoproteins C, D and E protects guinea pigs against HSV-1 genital infection. Hum Vaccin Immunother. 2020;16(9):2109–13. doi: 10.1080/21645515.2020.1749509 32347775 PMC7553673

[61] Weissman D, Pardi N, Muramatsu H, Karikó K. HPLC purification of in vitro transcribed long RNA. Methods Mol Biol. 2013;969:43–54. doi: 10.1007/978-1-62703-260-5_3 23296926

[62] Kaushic C, Ashkar AA, Reid LA, Rosenthal KL. Progesterone increases susceptibility and decreases immune responses to genital herpes infection. J Virol. 2003;77(8):4558–65. doi: 10.1128/jvi.77.8.4558-4565.2003 12663762 PMC152159

